# Recent Advances in Organelle-Targeted Fluorescent Probes

**DOI:** 10.3390/molecules26010217

**Published:** 2021-01-04

**Authors:** Na-Eun Choi, Ji-Yu Lee, Eun-Chae Park, Ju-Hee Lee, Jiyoun Lee

**Affiliations:** Department of Next-Generation Applied Science, and Global Medical Science, Sungshin University, Seoul 01133, Korea; naeunchoi@sungshin.ac.kr (N.-E.C.); 220206097@sungshin.ac.kr (J.-Y.L.); 20161550@sungshin.ac.kr (E.-C.P.); jhleehh@naver.com (J.-H.L.)

**Keywords:** fluorescence imaging, organelle-targeting, chemical probes

## Abstract

Recent advances in fluorescence imaging techniques and super-resolution microscopy have extended the applications of fluorescent probes in studying various cellular processes at the molecular level. Specifically, organelle-targeted probes have been commonly used to detect cellular metabolites and transient chemical messengers with high precision and have become invaluable tools to study biochemical pathways. Moreover, several recent studies reported various labeling strategies and novel chemical scaffolds to enhance target specificity and responsiveness. In this review, we will survey the most recent reports of organelle-targeted fluorescent probes and assess their general strategies and structural features on the basis of their target organelles. We will discuss the advantages of the currently used probes and the potential challenges in their application as well as future directions.

## 1. Introduction

Fluorescent chemical probes have been extensively used to study biochemical events within live cells. The advent of super-resolution imaging techniques and the availability of a wide variety of fluorescent probes enable effective subcellular tracking of transient metabolites and signaling molecules that are involved in important physiological processes. Probes targeting specific organelles such as mitochondria and lysosomes have been used routinely to monitor organelle functions and have become invaluable tools for the investigation of disease-relevant pathways. These probes efficiently target subcellular organelles; however, considering the complexity and diversity of biochemical processes inside the cells, probes that are not only specific to target organelles but also tailored to applications are highly desirable.

Each subcellular organelle has unique physicochemical and structural characteristics that can be utilized for organelle-specific targeting. For example, mitochondria have a negative membrane potential, which attracts positively charged moieties; lysosomes have an acidic vesicular structure wherein weakly basic compounds can accumulate. Thus, commercially available mitochondria- or lysosome-tracking dyes are cationic amphipathic or weakly basic. An additional targeting moiety can be incorporated to detect specific proteins and chemical species inside the organelles; however, the added moiety may affect cellular permeability as well as organelle specificity. Considering that each organelle plays a different function and mediates diverse pathways, the target of interest must be carefully chosen to provide meaningful observations.

In this review, we outline the general strategies and structural characteristics of organelle-targeted fluorescent probes and categorize the most recent examples of these probes on the basis of their target organelles. In particular, we will focus on specific and most up-to-date examples for the extensively studied organelles such as mitochondria and lysosomes, but also cover recent developments associated with much less explored organelles including the endoplasmic reticulum, Golgi-complex, melanosomes, and lipid droplets. We also provide an overview of the most prominent applications and advantages of organelle-targeted probes and discuss potential challenges and future directions.

## 2. Design Strategies and Recent Examples

### 2.1. Lysosome-Targeted Probes

Lysosomes are weakly acidic vesicles encasing many housekeeping proteins and enzymes that are responsible for degrading cellular proteins, lipids, and nucleic acids. Lysosomal processing is a highly dynamic operation involving multiple endocytic pathways and autophagy that are governed by lysosomal signaling [1]. In particular, lysosomal H^+^ and other ions such as Ca^2+^, Fe^2+^, Zn^2+^, and Cl^−^ are the key regulators of lysosomal function; impaired ion homeostasis can lead to defects in lysosomal trafficking and storage, which are associated with neurodegenerative diseases and metabolic disorders [2,3]. It has also been reported that lysosomal Ca^2+^ and Fe^2+^ are crucial for redox signaling in autophagy and crosstalk between lysosomes and mitochondria [4]. Hence, many studies focused on monitoring the oxidative and lytic functions of lysosomes by measuring lysosomal pH and redox-active chemical species. Commercially available lysosome-tracking dyes comprise a pH-sensitive core that generates fluorescent signals upon protonation. While these dyes effectively stain acidic compartments, dyes that can selectively localize and track various chemical species inside lysosomes are needed. In this section, we provide an overview of recently developed probes that can specifically target and monitor lysosomal chemical species and pH.

Cellular thiols, such as cysteine (Cys), homocysteine (HCy), glutathione (GSH), and hydrogen sulfide (H_2_S), are one of the common target species that have been investigated using lysosome-targeted probes. These biothiols are generated from lysosomal proteolysis and are prominent indicators of lysosomal function [5]. Zhang et al. reported a lysosome-targetable fluorescent probe that can monitor multiple thiol-species with different spectral output patterns [6]. They used a dual dye scaffold containing coumarin and resorufin to induce ratiometric signal changes upon reaction with thiols. Specifically, their probe, Lyso-RC (**1**), generated three different species in response to the reaction with H_2_S, Cys/HCy, and GSH, simultaneously sensing multiple thiols in live cells. For lysosomal targeting, they attached morpholine, a lysosomotropic amine, trapped in the environment of lysosomal pH (4.5–4.7) [7,8]. Li et al. reported a 1,8-naphthalimide-based lysosome-targeted probe that reacts with thiols, but generates a strong fluorescence signal only responding to H_2_S [9]. The BHNP-DA probe (**2**) contains a disulfide group that can rapidly react with thiols; however, it reacts only with the terminal thiol that is close to the ester linkage between the fluorophore and the disulfide adduct. In this case, H_2_S undergoes a cyclization reaction producing a strong fluorescence signal.

**Figure 1:**
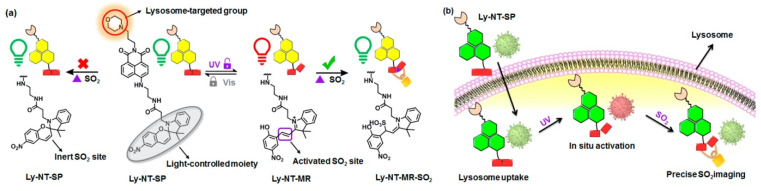
(**a**) Molecular design of **6** and proposed sensing mechanism; (**b**) Schematic illustration of in situ response of **6** controlled by UV irradiation. Reprinted with permission from ref. [13]. Copyright 2020 American Chemical Society.

Hypochlorous acid (HClO) and sulfur dioxide (SO_2_) are important markers of lysosomal function and oxidative stress; therefore, many probes have been designed to detect these redox-active species. Yuan et al. reported a coumarin–rhodamine conjugate for the ratiometric sensing of HClO [10]. The probe contains a monothio-bishydrazide linker that undergoes a rapid cyclization reaction with lysosomal HClO (**3**). Although they did not conjugate any lysosome-targeted group, the authors reported that the weakly basic monothio-bishydrazide moiety acted as a lysosomotropic group, selectively delivering the probe to the lysosomes. Zhang et al. also developed a two-photon fluorescent probe to detect HClO, but used a different approach [11]. A morpholine was conjugated with a fluorophore containing a redox-active methyl thioether (**4**). The probe had a strong emission at 505 nm, which decreased upon reaction with ClO^−^ because of the oxidation of the thioester; however, the fluorescence signals with decreased intensity were recovered by the subsequent addition of GSH, suggesting that the probe can be used to monitor the intracellular HClO/GSH redox cycle. Another strategy introduced by Ren et al. demonstrated that a photocaged probe containing a morpholine and a dibenzoylhydrazine group (**5**) can be effectively localized inside lysosomes and can release the reactive probe upon UV light illumination [12]. This strategy is useful for maximizing the lysosomal delivery of the probe, while minimizing off-target fluorescence signals. Yin et al. applied the same light-controlled detection strategy to monitor SO_2_ levels during a heat stroke in the intestinal tissues of mice [13]. They incorporated a light-responsive spiropyran group, which isomerizes into an activated merocyanine (Ly-NT-SP; **6**; Figure 1). They measured SO_2_ levels during the heat shock and observed that lysosomal SO_2_ acts as an antioxidant in response to oxidative stress. Combined with the light-controlled detection method and lysosome-specific targeting, these probes can be applied to investigate potential disease markers and disease pathology.

**Figure 2:**
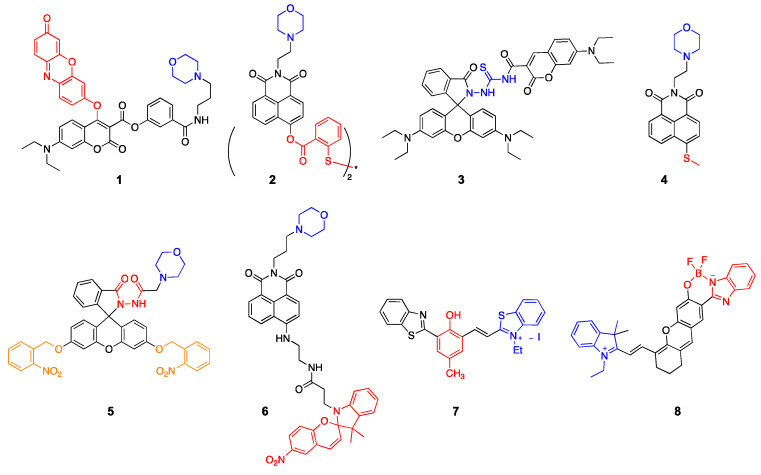
Lysosome-targeted fluorescent probes (blue: targeting moiety; red: responsive moiety; orange: photocaging group).

Lysosomal pH has been the prime target for many fluorescent probes that were extensively used to study lysosomal function and physiology. While these widely available probes are highly sensitive and easily accessible, they utilize weakly basic lysosomotropic agents that can affect lysosomal functions and cell viability via intra-lysosomal trapping [14,15]. Dahal et al. introduced a near IR (NIR)-emitting pH probe (**7**) based on a cyanine scaffold that can avoid lysosomal trapping [16]. The probe utilizes a reversible phenol/phenoxide interconversion for lysosome-specific fluorescence enhancement without increasing lysosomal pH. The probe also exhibited a large Stokes shift (234 nm) with an emission maximum at 700 nm, demonstrating favorable characteristics for in vivo imaging experiments. Shi et al. also reported a NIR boron complex to monitor lysosomal pH [17]. This probe, HCy-BIZ-BF2 (**8**), contains a pH-responsive boron complex in the cyanine scaffold, showing good photostability in living cells and animals with an emission maximum at 710 nm. These NIR probes can potentially be applied for in vivo diagnostic imaging. We outlined structures of all probes described in this section in Figure 2.

### 2.2. Nucleus-Targeted Probes

The cell nucleus has been a major target for cancer therapy and genetic engineering; it contains the genetic material enclosed by the nuclear envelope consisting of two lipid bilayer membranes. The nuclear envelope is a tightly regulated membrane barrier; thus, nucleus targeting was achieved either by passive diffusion or by active transport via the nuclear pore complex (NPC) [18]. While nucleus-targeted delivery methods have been extensively studied for therapeutic purposes, fluorescent probes targeting the nucleus have limited applications such as staining DNAs. These conventional DNA-binding dyes are mostly DNA intercalators and are used to detect and quantify nucleic acids. However, recently reported DNA-binding probes have more specific purposes; for example, Barton et al. developed a rhodium complex–cyanine conjugate (**9**) that can detect mismatched DNA [19]. The rhodium complex selectively inserts into structurally unstable mismatched DNA, and the conjugated Cy3 dye generates increased fluorescence because of the restricted rotation. The probe selectively detects a CC mismatch in genomic DNA samples in multiple cell lines although it was not tested in live cells for its potential to determine nucleus-specific accumulation. In their subsequent work, the pyridylalcohol-coordinated derivative Rh-O demonstrated nuclear and mitochondrial localization in live cancer cells likely via passive diffusion [20]. Tang and coworkers developed a fluorescent probe containing a benzothiazole scaffold (**10**) that can selectively bind to a DNA G-quadruplex [21,22]. Their probe, named IMT, has a structure similar to that of commercially available Thioflavin T (ThT), except that IMT has a *N*-isopropyl group in place of the *N*-methyl group of ThT, which binds the DNA G-quadruplex [23]. Unlike ThT, IMT only localizes in the nucleus because of the increased hydrophobicity resulting from the *N*-isopropyl substituent.

Bucevičius et al. developed rhodamine-Hoechst 33,258 conjugates (**11**) that stain DNA with enhanced brightness suitable for stimulated emission depletion (STED) imaging [24]. While Hoechst conjugates have been used for DNA-targeting, they found that 5′-and 6′-regioisomers of rhodamine-Hoechst conjugates have distinct spectroscopic properties and different DNA binding modes. These conjugates are highly photostable and bind to AT-rich heterochromatin regions, enabling super-resolution imaging of the heterochromatin dynamics in live cells. One of their conjugates, 5′-580CP-Hoechst was used to obtain high-resolution images of DNA and tubulin structures in intact animal erythrocytes, demonstrating practical applications for live cell imaging. Lämmle et al. reported a photocaged Hoechst dye (pcHoechst; **12**) which allows the spatiotemporal control of subnuclear DNA labeling [25]. Their probe was nontoxic for cells and zebrafish and specifically stained subnuclear DNA upon UV irradiation, suggesting that the probe can be used for not only chromosomal DNA, but also extranuclear DNA during viral entry events or tumor-specific mutations.

**Figure 3:**
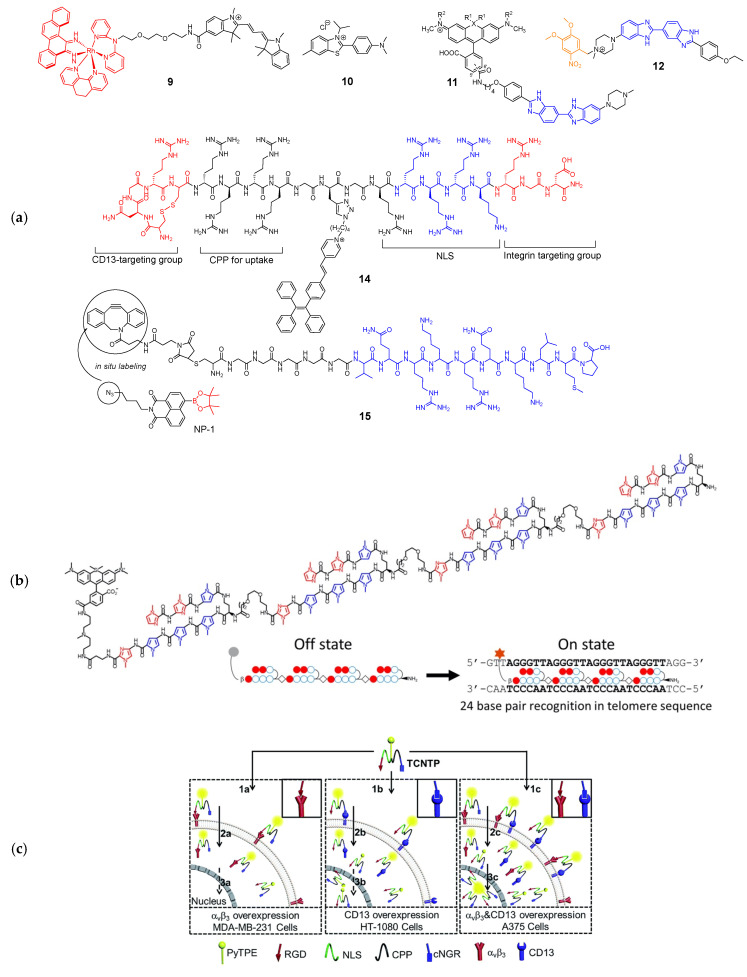
(**a**) Structures of nucleus-targeted probes (blue: targeting moiety; red: responsive moiety; orange: photocaging group); (**b**) Structure of **13** (SiR-TTet59B) and a schematic representation of fluorogenic recognition of the telomere sequence. Reprinted with permission from ref. [30]. Copyright 2020 American Chemical Society; (**c**) Schematic illustration of **14** targeting the nucleus of integrin α_ν_β_3_ and CD13-overexpressed cancer cells. Reprinted with permission from ref. [31]—Published by The Royal Society of Chemistry.

Pyrrole-imidazole (Py-Im) polyamides are known to bind specific sequences of DNA [26], and have been widely used as for the detection of specific DNA sequences [27] and the modulation of gene expression [28] and transcription [29]. Recently, Tsubono et al. reported a Py-Im polyamide-derived probe that can image telomeres in live cells [30]. The probe consists of a near-infrared emitting silicon-rhodamine fluorophore (SiR) and a tandem tetramer Py-Im polyamide (TTet59B, Figure 3) which specifically binds to telomeres and exerts enhanced fluorescence signals (**13**). It should be noted that **13** itself did not localize in the nuclei, and was accumulated in lysosomes unlike previously reported Py-Im polyamides, likely due to the large tetrameric structure. To circumvent this problem, cells were pretreated with a weakly basic peptide that can release the compounds trapped inside endosomes. While **13** achieved a highly specific binding to telomeres, an alternative strategy, such as a supramolecular assembly or structural simplification is needed to avoid endosomal entrapment for more broad applications.

In addition to passive targeting, peptide-based delivery utilizing nuclear localization sequence (NLS) has been studied. Several NLSs were reported, which commonly contain K-K/R-X-K/R sequences that can be actively imported through NPC. Cheng et al. reported a multifunctional probe targeting the nucleus of integrin α_ν_β_3_ and CD13-overexpressed cancer cells [31]. Although it is a single molecule, its probe has a CD13 targeting peptide, a cell-penetrating peptide, NLS, and RGD to maximize cellular uptake and tumor targeting (TCNTP; **14**). In particular, they used the AIEgen (aggregation-induced emission) to minimize the fluorescence quenching effect and to allow long-term tracing of cancer cells.

Yin and coworkers also used NLS for targeting, but applied a stepwise approach [32]. In their previous work, they observed that the simple combination of their H_2_O_2_ probe (NP-1) and NLS yielded only marginal nuclear uptake and speculated that the conjugation of the probe affected the interaction between NLS and importin, a nuclear transport protein subunit [33]. Therefore, to improve the nucleus-localization of the probe, they attached dibenzocyclooctene via a hexaglycine linker to NLS and modified NP-1 with an azide group for in situ click reaction (**15**). The co-treatment of the modified NLS (pep6) and NP-1-loaded cells showed a much improved nuclear uptake compared to that of their previously reported probe; however, it took 18 h for the optimal uptake and click reaction, which may not be suitable for real-time tracking of nuclear H_2_O_2_ in live cells under various physiological conditions. In this example, stepwise in situ labeling targeting the nucleus seems feasible, although it needs to be improved for practical use.

### 2.3. Membrane-Targeted Probes

The cell membrane is not an intracellular organelle; however, the recently discovered involvement of membrane microdomains (rafts) in viral infection [34,35] and cancer [36] and the likely contribution of the cell membrane to amyloid formation in neurodegenerative diseases [37,38] make it an important target for the investigation of cell membrane dynamics and morphology. Currently available membrane-targeted probes share a common approach—conjugation of an environment-sensitive fluorophore to generate membrane-specific signals and a membrane-anchoring moiety to minimize diffusion of the probe. Recently reported probes incorporate additional moieties to increase the number of fluorescence signals for high-resolution imaging or to detect intracellular signaling molecules. Xu and coworkers developed a membrane probe that can self-assemble in the plasma membrane triggered by GPI-anchored ectophosphatase [39]. Their probe, 1P (**16**), consists of three parts: Environmentally sensitive 4-nitro-2,1,3-benzoxadiazole (NBD) fluorophore, membrane-anchoring cholesterol, and self-assembly triggering phospho-D-tyrosine. The phosphor-D-tyrosine groups are hydrolyzed upon contact with membrane ectophosphatases, inducing the self-assembly of the probe in the plasma membrane and revealing the heterogeneous distribution of lipid rafts. In particular, **16** visualized changes in the membrane dynamics of cancer cells upon treatment with an anti-cancer drug candidate, demonstrating its potential application in drug screening.

**Figure 4:**
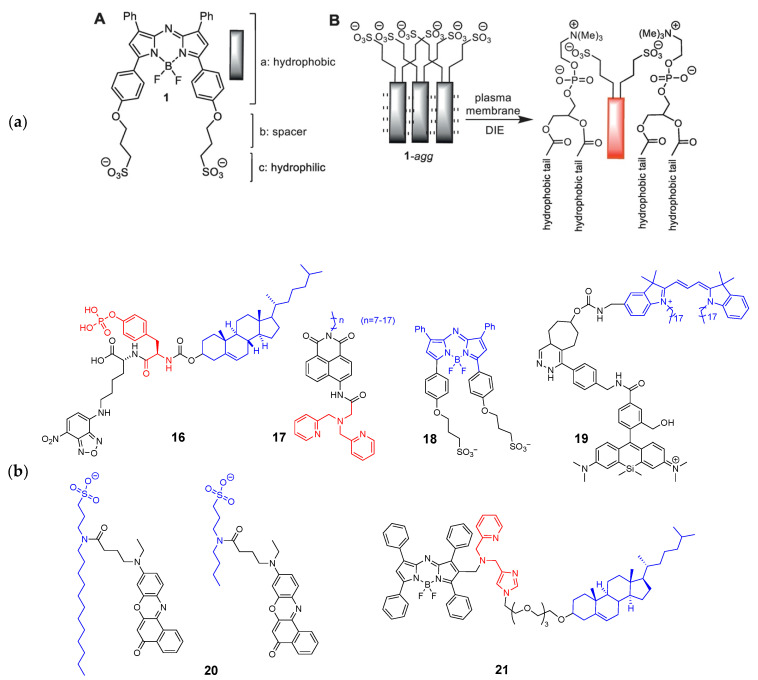
(**a**) A: Amphiphilic NIR-AZA probe (**18**); B: Simplified schematic showing representation of membrane DIE activation of aggregated **18**. Reprinted with permission from ref. [41], copyright Elsevier 2018; (**b**) Structures of membrane-targeted probes (blue: targeting moiety; red: responsive moiety).

Deng et al. used an opposite approach to increase the fluorescence signals. They designed a membrane-targeted Zn^2+^ that can monitor Zn^2+^ release from living cells over a period of time [40]. Their probe consists of a hydrophobic alkyl chain (carbon chain lengths from 8 to 18) and an NBD fluorophore that has an additional reporter group, dipicolyl amine, for Zn^2+^ sensing (**17**). Because of the amphipathicity, the probe forms a micelle that quenches fluorescence; however, once the micelle interacts with the plasma membrane, the micellar particle dissociates into individual probes. The alkyl chain of the dissociated probes now acts as an anchoring group and the probe can selectively react with extracellular zinc ions. O’Shea et al. also followed a similar approach to increase the fluorescence signals by using a disaggregation-induced emission (DIE)-responsive probe [41]. They developed NIR-emitting aza-BODIPY with bis-sulfonic acid substituents (NIR-AZA, **18**). The probe is amphiphilic and prone to aggregation in aqueous environment, becoming non-fluorescent. However, once the aggregates contact the plasma membrane, the hydrophobic aza-BODIPY core is inserted into the membrane lipid, while the bis-sulfonic acid groups interact with surface residues, resulting in disaggregation and fluorescence emission enhancement (Figure 4). It is noteworthy that unlike other membrane-targeted probes, NIR-AZA utilizes its amphipathic nature by incorporating hydrophilic groups instead of hydrophobic anchoring groups.

Takakura et al. developed a set of fluorescent probes with blinking property, termed HIDE (high-density, environment-sensitive) membrane probe [42]. They utilize a silicon-rhodamine dye that shows on/off fluorescence depending on hydrophobic environment (**19**). The blinking property is particularly important for super-resolution microscopy, because the technique relies on the precise localization of single-molecule emitters [43]. By conjugating various membrane-targeting group via in situ click chemistry, they acquired super-resolution images of the plasma membrane, mitochondria, and endoplasmic reticulum. In their following study, they used a carborhodamine in place of a silicon-rhodamine, achieving much better photostability and two-color time-lapse imaging capability [44]. Danylchuk et al. reported a series of membrane-targeting switchable probes containing a solvatochromic dye Nile Red [45]. In this work, the authors introduced an alkyl chain with a sulfonate group which can control the membrane-binding affinity (**20**). The probe with a long alkyl chain, NR12A, binds to plasma membrane irreversibly and exerts intense fluorescence in response to lipid density, which is suitable property for traditional microscopy. The probe with a short alkyl chain, NR4A, reversibly binds to the membrane and generates continuous blinking of the probe, enabling super-resolution imaging of membrane topology.

Most studies discussed in this review utilize previously reported targeting groups and focus on the applications in live cells rather than the fate or biological effects of the probes themselves. However, Kim et al. reported that the membrane targeting the cholesterol group affected the membrane integrity and probe permeability [46]. The reported probe (JJ, **21**) consists of three units: An aza-BODIPY fluorophore, a zinc ion sensing group, and a cholesterol moiety connected via a triethylene glycol (TEG) linker. The probe exhibited highly selective turn-on signals in response to Zn^2+^ when it was treated with HeLa cells, but the probe did not stay in the membrane and rapidly internalized in the lysosomes and endoplasmic reticula together with the exogenous zinc ions. The probe without the TEG-cholesterol group did not exhibit membrane permeability and showed only extracellular fluorescence; therefore, the authors proposed that TEG-cholesterol might be responsible for altered membrane permeability. Considering that cholesterol is a crucial component of lipid rafts and affects membrane lipid packing and permeability [47,48], the physiological impact of the probes must be carefully monitored and evaluated.

### 2.4. Mitochondrion-Targeted Probes

Mitochondria play critical roles in cell physiology, including ATP production, oxidative respiration, and calcium-mediated signal transduction. Moreover, many mitochondrial pathways are directly associated with metabolic disorders [49], cancer [50], and neurodegeneration [51]. The mitochondrion is the center of cellular respiration and an energy-producing hub, releasing reactive oxygen species (ROS), such as hydrogen peroxide (H_2_O_2_) and hypochlorite (ClO^−^), which can act as a crucial signaling molecule and can cause cell damage [52]. Therefore, probes targeting mitochondrial ROS have been extensively used to study many disease-relevant mitochondrial pathways [53]. Because of the unique mitochondrial structure, having a double-layered membrane with a negative membrane potential, mitochondria-targeted probes require a positively charged scaffold that is also highly hydrophobic [54]. Compared to other organelle-targeted probes, mitochondria-targeted probes probably have the most diverse structures because many fluorophores have cationic and lipophilic characteristics, which can serve as a mitochondria-targeting group without introducing additional moieties. Tetramethylrhodamine- and benzothiazole-based fluorophores are used most frequently, but fluorophores that can be readily modified to contain basic amine groups have been evaluated for mitochondria targeting.

He et al. reported a ratiometric fluorescent H_2_O_2_-detection probe based on a 2-(2′-hydroxyphenyl) benzothiazole (HBT) scaffold equipped with a boronate ester group [55]. The boronate ester group was conjugated to the HBT core via a quinoline ring as a bridge, which also serves as a positively charged mitochondria-targeting group (**22**). The boronate ester is hydrolyzed upon reaction with H_2_O_2_, and the quinoline ring is cleaved subsequently, leaving only the HBT core inside the mitochondria. The probe itself absorbs at 564 nm and emits at 666 nm, but once the reactive boronate ester is removed, the remaining fluorophore absorbs at 340 nm and emits at 594 nm, which enables ratiometric imaging for H_2_O_2_ quantification. Tang et al. also introduced a NIR probe (**23**) by applying a similar strategy, but in their probe, they placed the reactive boronate ester into the hydroxyphenyl group, which generates turn-on signals upon hydrolysis [56].

Hu et al. developed a far-red-emitting ratiometric fluorescent probe to detect hypochlorite (ClO^−^) in cancer cells (**24**) [57]. While most H_2_O_2_-detecting probes utilize a reactive boronate ester, probes designed to detect ClO^−^ have an ethylene group that undergoes rapid oxidation to generate an aldehyde. Hu et al. used a charged hemicyanine group as a mitochondrion-targeting moiety as well as an electron donor group that is cleaved upon oxidation and produces a ratiometric fluorescent emission change. Zhu et al. tested a well-known DNA staining dye, Nile Blue, for use as a mitochondrion-targeted hypochlorite probe (**25**) [58]. The aniline group in Nile Blue undergoes rapid oxidation (<5 s) upon reaction with hypochlorite. Nile Blue has been used to stain frozen tissue sections and in-gel DNAs. When it is applied in live HeLa cells, it is localized in the mitochondria; however, it has been also reported that Nile Blue accumulates in the lysosomes of tumor cells [59] therefore, a thorough mechanistic study of its uptake may be needed.

**Figure 5:**
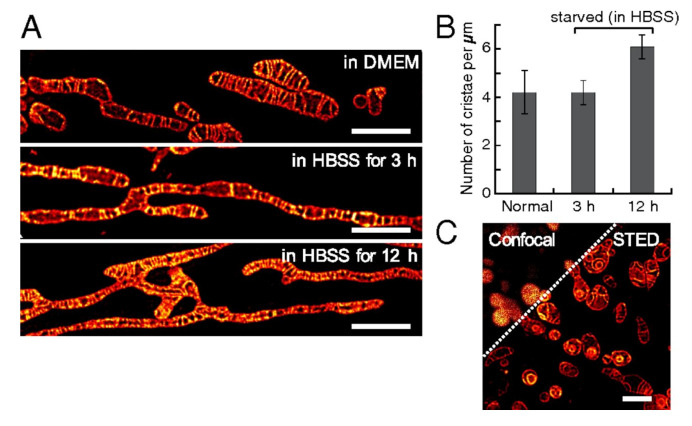
Morphological changes of the mitochondrial inner membrane captured by S stimulated emission depletion (STED) microscopy using **26**. (**A**): Deconvoluted STED images showing changes in the mitochondrial morphology; (**B**): Comparison of the number of cristae per micrometer of mitochondrial length before and after incubation for 3 and 12 h under starvation conditions; (**C**): STED image of cristae in HeLa cells, pretreated with 10 µM mitochondrial DNA replication inhibitor (ddC) for 5 days followed by staining with MitoPB Yellow. Reprinted with permission from ref. [62].

Recent advances in super-resolution microscopy enabled the imaging of cellular organelles in as much detail as electron microscopy. One of the critical factors to consider for successful super-resolution imaging is the use of high contrast and photostable dyes that can reduce exposure time and tolerate high-energy laser irradiation [60]. Although more stable and brighter fluorophores have been designed and tested, a more effective way to overcome the limitations of conventional dyes is to maximize local dye concentrations. For mitochondria, lipophilic cations such as triphenylphosphonium (TPP) groups can be conjugated to enhance the local delivery of probes [61]. Yamaguchi et al. introduced one such example, a photostable fluorescent probe for super-resolution live cell imaging of mitochondria [62]. In their work, they developed a novel naphthophosphole *P*-oxide fluorophore (**26**) for stimulated emission depletion (STED) microscopy. The probe is water soluble, but weakly fluorescent in solution; thus, a TPP group and an epoxide group were additionally conjugated to maximize mitochondrial localization. Time-lapse STED imaging of live HeLa cells using the probe clearly demonstrated inter-mitochondrial fusion and mitochondrial ultrastructure (Figure 5), suggesting that the mitochondria-targeted fluorophores can be used for high-resolution imaging of single organelle dynamics.

Another example of using TPP as a mitochondria-targeting group, reported by Matile et al., showed that the conjugation of TPP selectively delivered a mitochondrial tension probe without affecting the mitochondrial function [63]. In their previous work, the Matile group developed planarizable push-pull probes named “fluorescent flippers”, inspired by oligothiophenes that provide blue-shifted excitations upon ring twists. These fluorescent flippers respond to mechanical reorganization of lipid bilayers resulting in red-shifted excitations [64]. Their novel probe designs incorporated a mitochondria-targeting group (TPP), a lysosome-targeting group (morpholine), and an ER-targeting alkyl chain. Mito-flippers (**27**) were used for fluorescence lifetime imaging microscopy and detected mitochondrial membrane tension changes upon osmotic shock. This work again demonstrated that mitochondrion-targeted fluorescent probes are excellent tools for studying membrane dynamics.

**Figure 6:**
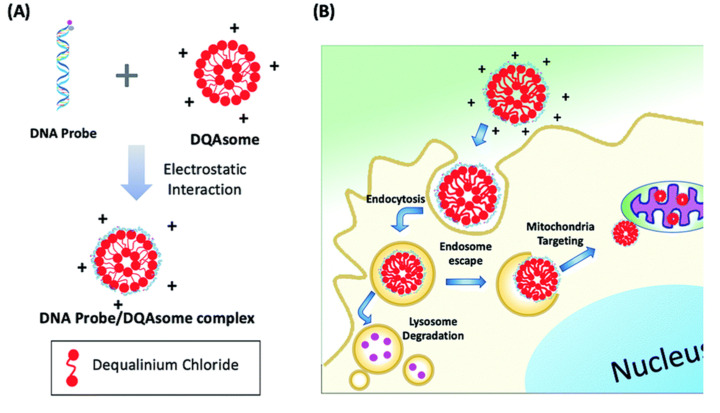
(**A**) Aptamer/DQAsome-based mitochondrion-targeted probe (**28**) and schematic of DNA probe/DQAsome complex formulation. (**B**) Schematic depiction of targeted delivery of DNA probe/DQAsomes to mitochondria. Reprinted with permission from ref. [65]—Published by The Royal Society of Chemistry.

For specific targeting of fluorescent probes, polymer-based delivery systems have been also used. Hong et al. developed a photocaged aptamer-based ATP sensor (**28**) that is delivered to the mitochondria using liposome-based polymeric transporters (Figure 6) [65]. Because of their high selectivity and sensitivity achieved by performing multiple selection cycles, aptamer-based probes and reagents were in demand with respect to various applications in recent years [66,67,68]. The major disadvantages of aptamer-based probes are their relatively high molecular weight (5–15 kDa) and metabolic instability with high polarity, which can be overcome by a suitable delivery method for enabling effective intracellular applications. To this end, Hong et al. used DQAsomes (dequalinium-based liposome-like vesicles [69]) for the mitochondria-targeted delivery of aptamer-based probes. Their photo-cleavable aptamer sensor (PC-Apt, **28**) is partially hybridized with a short complementary sequence to block ATP binding; however, upon light irradiation (365 nm), the short complementary sequence is cleaved and exposes the ATP binding region. The ATP-bound aptamer subsequently folds into an active conformation and releases a fluorescence quencher fragment and exerts enhanced fluorescent signals. Using the mitochondrion-targeted DQAsome, the probes selectively accumulated inside the mitochondria and successfully detected ATP only upon light irradiation. This probe demonstrates that introducing a photocleavable group and an organelle-specific targeting group enables the spatiotemporal control of a fluorescent probe with high sensitivity.

**Figure 7:**
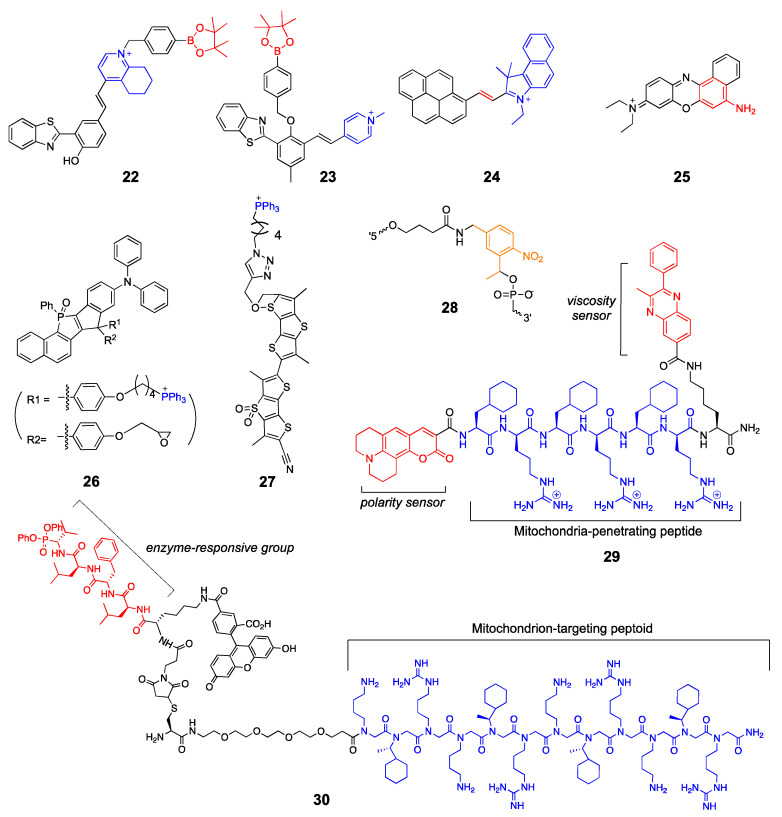
Mitochondrion-targeted probes (blue: targeting moiety; red: responsive moiety; orange: photocaging group).

Peptide- and peptidomimetics-based transporters are also useful for the mitochondria-targeted delivery of fluorescent probes. Kelley et al. developed a multifunctional chemical probe to detect microviscosity and micropolarity changes in the mitochondria [70]. The probe (**29**) has a mitochondria-penetrating peptide sequence, viscosity-sensing phenylquinoxaline, and polarity-dependent coumarin 343. The probe visualized changes in mitochondrial viscosity and polarity upon treatment with ionophores and electron transport complex inhibitors. Nam et al. used a peptoid-based mitochondria-targeting group that is conjugated to an activity-based probe (**30**) to label the active mitochondrial enzyme HTRA2 (the high-temperature requirement A) serine protease in live cells [71]. This probe is particularly useful for monitoring enzyme activity changes in living cells without the use of cell permeabilizing agents or multiple antibodies and is applicable for diagnostic imaging. Structures of the mitochondrion-targeting probes described in this section can be found in Figure 7.

### 2.5. Probes Targeting the Endoplasmic Reticulum (ER) and Golgi Apparatus

In cellular physiology, newly produced proteins and lipids are transported from the ER to the Golgi apparatus. The ER, Golgi, lysosomes, and the cell membrane are closely connected and often referred to as “the secretory pathway”, where proteins and lipids are sorted and distributed to other organelles or secreted into the extracellular environment [72]. Considering the importance of the secretory pathway in protein biogenesis and quality control, genetically encoded fluorescent proteins and specific imaging techniques have been developed to visualize the dynamics of the ER-Golgi transport [73]. As we have seen in the previous sections, chemical probes are extensively used for other organelles; however, ER- and Golgi-targeted fluorescent probes are rarely reported in literature mainly because specific targeting mechanisms have not been elucidated. Similar to the mitochondrial targeting sequence (MTS) and nuclear localization signal (NLS), ER- and Golgi-targeting sequences have been reported [74,75]; however, these sequences have large molecular weights because they consist of approximately 100 amino acids, making them unsuitable for intracellular delivery. Small molecules such as brefeldin A and rapamycin are also known to localize in the ER and Golgi network [76]; however, they have intrinsic pharmacological activity and are also inapplicable for targeting purposes. The most widely used ER- and Golgi-targeting moiety is the phenyl sulfonamide group that selectively binds to cyclooxygenases (COX) that are abundant in the ER membrane [77]. Thus, probes in this category share a similar scaffold—a phenylsulfonamide group conjugated to a fluorophore that can detect chemical species in the ER and Golgi complex. Both the ER and Golgi stress responses are implicated in neurodegenerative diseases such as Alzheimer’s disease, Amyotrophic lateral sclerosis (ALS), and Huntington’s disease [78,79]. These targeted probes mostly focus on monitoring cellular levels of stress-responsive chemicals, including NO, H_2_S, and HOCl.

Li et al. developed an ER-targeted two-photon probe to detect cellular NO levels under ER stress [80]. The probe (**31**) has an ER-targeting *p*-toluenesulfonamide group, a naphthalimide fluorophore, and an *o*-phenylenediamino group for the selective detection of NO. Confocal images of the cells treated with **31** in the presence of exogenous NO showed a strong correlation between the probe and ER, whereas a poor overlap with mitochondria and lysosomes was observed, indicating that the probe is specifically localized in the ER. The probe also exhibited enhanced fluorescence signals in response to the treatment of the ER stress inducer, tunicamycin, and was used to detect NO in tunicamycin-treated mice. It should be noted that the phenylsulfonamide group can bind to the Golgi complex as well as the ER; however, Li et al. did not mention any potential cross-reactivity and acknowledged that the probe could freely diffuse into the cytosol and react with intracellular NO regardless of their location.

Zhu et al. recently developed a Golgi-targeted fluorescent probe that can detect endogenous H_2_S in cells and zebrafish under Golgi stress response [81]. Their probe, Gol-NH (**32**), has a scaffold similar to that of the ER-targeted **31**, except that **32** has an azide-containing naphthalimide fluorophore for H_2_S detection. Based on the fluorescence imaging experiments, the probe appears to selectively localize with the Golgi complex (correlation coefficient *r* = 0.92), whereas it localizes to a lesser extent with other organelles such as lysosomes, ER, and mitochondria (*r* = 0.58, 0.45, 0.52, respectively). In their experiments, Golgi-specific stress inducers, such as nigericin and brefeldin A, were used to treat cells; hence, only the Golgi-stress-induced signals were observed. Considering that the phenyl sulfonamide group preferentially localizes to the ER membrane, additional reagents to suppress the signals from other organelles may be needed. Indeed, the probe showed strong fluorescence even when exogenous H_2_S was added to zebrafish, suggesting that **32** responds to intracellular H_2_S, but not necessarily to Golgi-specific H_2_S.

**Figure 8:**
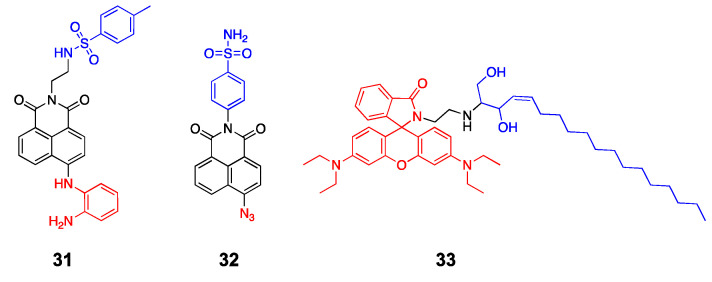
Endoplasmic reticulum (ER)- and Golgi-targeted probes (blue: targeting moiety; red: responsive moiety).

Fan et al. used a different approach to target the Golgi apparatus [82]. Commercially available fluorescent trackers for the Golgi apparatus contain ceramides and sphingomyelins that can effectively serve as a structural marker for the *trans*-Golgi network [83]. They incorporated a sphingosine group with a pH-sensitive rhodamine B dye to detect Golgi-specific pH changes. Their probe (**RSG**, **33**, Figure 8) undergoes a considerable fluorescence enhancement upon a pH change from 7.4 to 2.0 and selectively localizes to the Golgi complex in live cells. They also induced oxidative stress conditions by using H_2_O_2_ and *N*-ethylmaleimide and observed Golgi-specific pH changes. While **33** demonstrated fluorescence enhancement in response to Golgi-specific pH changes in cells and animals, the pKa value of **33** is approximately 4.4, thus limiting its detection range from 6.0 to 3.0. Fine-tuning the scaffold with various substituents may be required for more practical applications in the future.

### 2.6. Probes Targeting Other Organelles

In addition to the major cellular organelles, eukaryotic cells have various transient organelles, such as peroxisomes, autophagosomes, and melanosomes. They are highly dynamic, interacting with other major organelles in response to various metabolic signals. Because they are often associated other organelles, such as mitochondria and lysosomes, and have similar properties, specific targeting is considered to be a challenging task. Probes targeting melanosomes have been relatively well studied, utilizing the melanosomal tyrosinase (TYR) [84] for targeting. Because melanosomes have acidic matrix similar to lysosomes, the melanosome-targeted probes have two targeting moieties; a morpholine group and a TYR-reactive group. Peng et al. reported a melanosome-targeting NIR probe (**34**) consisting of a morpholine, TYR-reactive *m*-hydroxybenzyl group, and a salicyladazine fluorophore [85]. The probe accumulates inside melanosomes and reacts with TYR, generating a strong fluorescence signal in melanoma cells. It also demonstrated TYR activity-dependent signals in various cells, enabling quantitative assessment of the TYR activity in living cells. Park et al. used a similar approach, except having a naphthalimide-based fluorophore for ratiometric imaging (**35**) [86]. Due to the utility of TYR for a potential biomarker in melanoma and other skin conditions, melanosome-targeted probes mostly contain a TYR-responsive group. While there are fluorescent proteins and antibodies specifically targeting the melanosomes regardless of TYR activity, chemical probes that can selectively stain melanosomes have not yet been reported.

Peroxisome is a small sized (0.1–1 μm) single membrane organelle encasing oxidative enzymes that generates and breaks down cellular hydrogen peroxide and reactive oxygen species [87]. The peroxisomal targeting signal 1 (PTS1) peptide is known to deliver oligomeric proteins into the peroxisomal matrix, and it is a short tripeptide S-K-L [88] making it an easily accessible targeting moiety for probe development. But, other than the first PTS1-BODIPY conjugated probe (**36**) reported two decades ago [89], fluorescent probes staining peroxisomes have not been reported to date. Given the involvement of peroxisomes in lipid metabolism and cellular respiration, peroxisome-targeted probes can be a powerful tool especially accompanied with high-resolution imaging techniques.

Autophagosome is a central machinery for autophagy, a cellular degradation process for cellular material turnover [90]. The formation of autophagosome starts with phagophore formation involving complex signaling processes, and eventually the autophagosome fuses with the lysosome to become the autolysosome. Because autophagy is a complicated process involving multiple pathways, many fluorescent probes have been developed to monitor autophagy; however, these probes are not necessarily target the autophagosomes, rather they mostly detect the changes in the lysosomal pH or viscosity [91]. Similar to other organelles in this section, fluorescent proteins and antibodies targeting autophagosomes have been widely used [92], however, chemical probes that can stain the autophagosomes selectively have not been reported.

**Figure 9:**
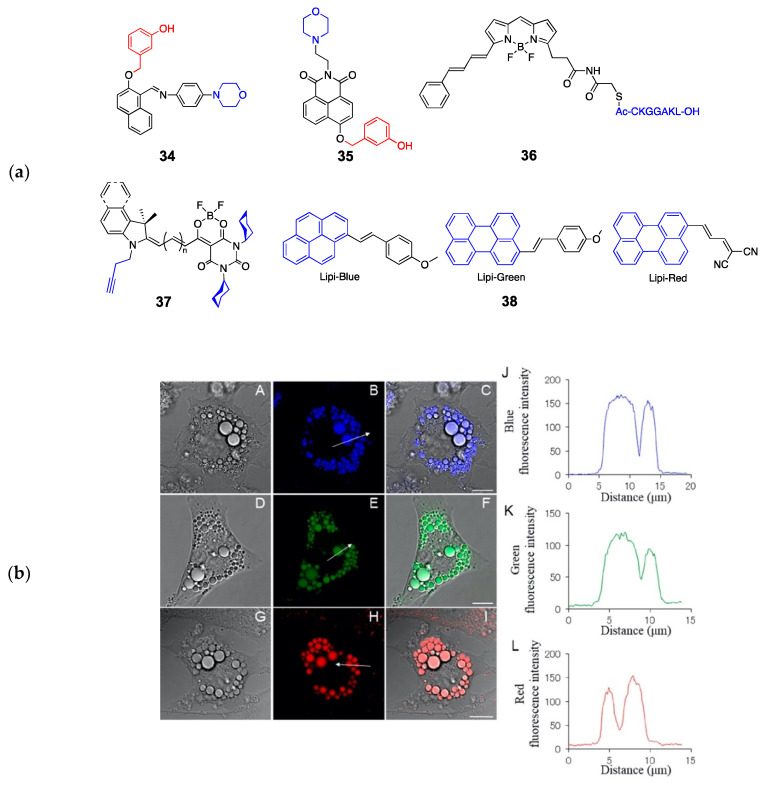
(**a**) Structures of probes targeting melanosomes (**34**–**35**), peroxisomes (**36**), and lipid droplets (LD) (**37**–**38**) (blue: targeting moiety; red: responsive moiety); (**b**) Fluorescence imaging of brown adipocytes stained with Lipi-probes (**38**)—Lipi-Blue (A–C), Lipi-Green (D–F), Lipi-Red (G–I). Images of differential interference contrast. (B, E, and H) Images of fluorescence. (C, F, and I) Images of overlay. The graphs on the right are results of the line-scan analysis: B, Lipi-Blue; E, Lipi-Green; or H, Lipi-Red. (J, K, and L) Fluorescence intensity of the range indicated by the arrows shown in parts B, E, and H, respectively. Scale bar is 10 μm. Reprinted with permission from ref. [97]. Copyright 2019 American Chemical Society.

Lipid droplets (LD) are lipid storage organelles containing a neutral lipid core surrounded by a single membrane. Recent studies indicate that LD are highly dynamic, interacting with different cellular organelles and regulating lipid metabolism [93]. Solvatochromic dyes such as Nile Red and dansyl-based fluorophores can be used as LD stains, but these dyes also stain the cell membrane, lacking specificity. Yang et al. found that the addition of a hydrophobic chain to the dansyl fluorophore provided sufficient specificity to target the neutral lipid core of LD [94]. Since then, many probes selectively targeting LD have been developed [95]. Collot et al. developed highly photostable and bright merocyanine dyes modified with cyclohexyl and pentynyl substituents for LD targeting [96]. Their probes (**SMCy**, **37**) generally exhibited high quantum yields (Φ = 0.2 − 0.68) and LD selectivity, enabling various imaging techniques such as 3D confocal imaging, multicolor imaging, and two-photon imaging. They also observed LD exchange between live KB cells. The presence of an alkyne side chain also allows click chemistry, easily turning the probe into a mitochondria-targeting probe. Tatenake et al. reported a pyrene/perylene-based LD-specific probes (**38**) with three different fluorescence emissions for multicolor imaging (Figure 9) [97]. In particular, they stained cells with different probes at different timepoints to investigate LD formation and trafficking, and successfully observed intercellular LD transfers.

## 3. Conclusions and Outlook

In this review, we discussed recent developments in organelle-targeted fluorescent probes. While most of the reported probes demonstrated excellent organelle specificity as well as high sensitivity, as shown in Table 1, only a few targeting moieties and detecting analytes have been extensively studied. This trend is prevalent not only in organelle-targeted probes but also in the field of fluorescent probe research wherein a wide range of fluorophores with different spectroscopic properties have been developed. Only a limited number of analytes, however, have been the subject of interest, such as ROS, metal ions, and pH. These probes are mostly based on modular design, consisting of fluorophores, targeting groups, and detection moieties. Therefore, different combinations of each component would easily generate new probes. Instead of switching fluorophores, introducing more diverse detection moieties and targeting groups may produce probes with higher efficiency, which will have more practical and broad-scale applications rather than serving as a proof-of-concept example. Considering that highly photostable and target-specific fluorescent probes are particularly useful in single molecule spectroscopy [98] and intra-operative imaging techniques [99], organelle-targeted fluorescent probes hold promise in both basic research and clinical fields. Novel probe designs overcoming the conventional modular approaches by incorporating photocaging groups or stepwise labeling methods are actively developed. These distinct strategies will facilitate the development of highly specific and sensitive fluorescent probes in the near future.

## Figures and Tables

**Table 1:** Summary of organelle-targeted fluorescent probes.

Entry	Organelle	Targeting Moiety	Detecting Analytes	λ_ex_/λ_em_ (nm)	Ref.
1	Lysosome	morpholine	H_2_S Cys/HCy GSH	580/602 (H_2_S) 376/480 (Cys/HCy) 438/540 (GSH)	[6]
2	Lysosome	morpholine	H_2_S	410/550	[9]
3	Lysosome	monothio-bishydrazide	HClO	410/480	[10]
4	Lysosome	morpholine	HClO	405/505	[11]
5	Lysosome	morpholine	HClO	480/525	[12]
6	Lysosome	morpholine	SO_2_	450/535	[13]
7	Lysosome	benzothiazolium	pH	415/694	[16]
8	Lysosome	hemicyanine	pH	635/730	[17]
9	Nucleus	rhodium complex	mismatched DNA	520/570	[19]
10	Nucleus	benzothiazole	DNA G-quadruplex	405/635	[21]
11	Nucleus	Hoechst	AT-rich region in DNA	352/455	[24]
12	Nucleus	Hoechst	AT-rich region in DNA	355/455	[25]
13	Nucleus	Py-Im polyamide	telomeres	645/665	[30]
14	Nucleus	NLS ^a^	integrin and CD13	450/560	[31]
15	Nucleus	NLS ^a^	H_2_O_2_	353/551	[32]
16	Membrane	cholesterol	membrane structure	465/550	[39]
17	Membrane	alkyl chain (C = 8–18)	Zn^2+^	405/525	[40]
18	Membrane	bis-sulfonic acids	membrane structure	700/720	[41]
19	Membrane	alkyl chain (C = 17)	membrane structure	642/700	[42]
20	Membrane	Nile Red	lipid composition	540/620	[45]
21	Membrane	cholesterol	Zn^2+^	622/663	[46]
22	Mitochondria	quinoline	H_2_O_2_	340/594	[55]
23	Mitochondria	*N*-methylpyridine	H_2_O_2_	405/669	[56]
24	Mitochondria	hemicyanine	ClO^−^	455/632	[57]
25	Mitochondria	Nile Blue	ClO^−^	600/672	[58]
26	Mitochondria	TPP ^a^	mitochondrial ultrastructure	488/(λ_STED_) 660	[62]
27	Mitochondria	TPP ^a^	mitochondrial membrane tension	430/570	[64]
28	Mitochondria	DQAsome	ATP	530/565	[65]
29	Mitochondria	mitochondria-penetrating peptide	mitochondrial polarity and viscosity	320/485 (polarity) 320/550 (viscosity)	[70]
30	Mitochondria	mitochondria-targeting peptoid	mitochondrial serine protease activity	494/512	[71]
31	ER	*p*-toluenesulfonamide	NO	440/538	[80]
32	Golgi	phenylsulfonamide	H_2_S	440/550	[81]
33	Golgi	sphingosine	pH	577/600	[82]
34	Melanosome	m-hydroxybenzyl	tyrosinase activity	500/675	[85]
35	Melanosome	3-hydroxybenzyl	tyrosinase activity	405/460	[86]
36	Peroxisome	acetyl-CKGGAKL	peroxisome biogenesis	530/550	[89]
37	LD	cyclohexyl	neutral lipids	526-770/550-794 (multicolor)	[96]
38	LD	pyrene, perylene	neutral lipids	381-520/445-650 (multicolor)	[97]

^a^ Abbreviations: NLS, nuclear localization signal; TPP, triphenylphosphonium; LD, lipid droplets.
